# Genetic Epidemiology in Latin America: Identifying Strong Genetic Proxies for Complex Disease Risk Factors

**DOI:** 10.3390/genes11050507

**Published:** 2020-05-04

**Authors:** Carolina Bonilla, Lara Novaes Baccarini

**Affiliations:** 1 Departamento de Medicina Preventiva, Faculdade de Medicina, Universidade de São Paulo, São Paulo 01246, Brazil; 2 Faculdade de Saúde Pública, Universidade de São Paulo, São Paulo 01246, Brazil; novaesbacca@gmail.com

**Keywords:** Latin America, genetics, Mendelian randomization, instrumental variables, FTO, rs9939609, Brazil

## Abstract

Epidemiology seeks to determine the causal effects of exposures on outcomes related to the health and wellbeing of populations. Observational studies, one of the most commonly used designs in epidemiology, can be biased due to confounding and reverse causation, which makes it difficult to establish causal relationships. In recent times, genetically informed methods, like Mendelian randomization (MR), have been developed in an attempt to overcome these disadvantages. MR relies on the association of genetic variants with outcomes of interest, where the genetic variants are proxies or instruments for modifiable exposures. Because genotypes are sorted independently and at random at the time of conception, they are less prone to confounding and reverse causation. Implementation of MR depends on, among other things, a strong association of the genetic variants with the exposure, which has usually been defined via genome-wide association studies (GWAS). Because GWAS have been most often carried out in European populations, the limited identification of strong instruments in other populations poses a major problem for the application of MR in Latin America. We suggest potential solutions that can be realized with the resources at hand and others that will have to wait for increased funding and access to technology.

## 1. Introduction

Mendelian randomization (MR) is a relatively new epidemiological method that aims to establish causal relationships between complex diseases and their risk factors. Since being described ~17 years ago [1], it has become more popular thanks to the availability and reduced costs of genome-wide genotyping techniques. MR relies on genetic variants that are strongly associated with risk factors of interest, usually identified via genome-wide association studies (GWAS), and assesses the association of these variants with disease (or other trait) outcomes. The association of the genetic variants with the outcome in proportion to their association with the risk factor or exposure indicates that the exposure might be causally associated with the outcome and provides an estimate of the causal effect. The use of genetic variants as proxies for the exposure, in what is known as the instrumental variable approach in econometrics, offers an innovative way to tackle some of the problems of observational epidemiological studies, in particular confounding and reverse causation [1]. Moreover, MR can be used to answer questions that cannot be addressed with randomized controlled trials (RCTs), due to ethical issues, implementation problems, or high costs. MR findings are also useful to avoid carrying out unnecessary RCTs, inform the design of future RCTs, and anticipate their results [2,3,4]. Nowadays, numerous MR extensions have been developed, most of which are described in a recently published dictionary [5].

The majority of MR studies to date have been carried out in European populations, whereas their implementation in Latin American populations has been particularly lacking, although recently, a couple of pioneering undertakings were reported [6,7]. Part of the reason for this is that the genetic variants used as proxies or instrumental variables (IVs) have been identified in Europeans, but their validation in non-Europeans has not been comprehensively accomplished. In addition, the difficulties of executing large-scale GWAS pose a significant drawback for the discovery of population-specific IVs.

Here, we examine the routes that could lead researchers working on Latin American populations to the identification of strong IVs for modifiable exposure underlying the regional risk for complex diseases. A combination of the routes explored is likely to be the best approach to achieve the goal of setting the stage for a systematic implementation of MR in Latin American populations.

## 2. Contributing Studies to Multi-Ethnic/Trans-Ethnic Meta-Analyses

These are analyses that combine data from local studies and are not restricted to one continental population. For instance, the 1982 Pelotas cohort from Brazil has been part of a number of so-called multi-ethnic, multi-ancestry, or trans-ethnic GWAS (for example, see references [8,9,10,11]), where information on European and non-European populations is pooled under the assumption that common single nucleotide polymorphisms SNPs driving complex trait associations are shared across diverse populations and that allele effects have concordant directions [12]. Given the large sample sizes and concomitant increased statistical power, this approach presents an opportunity to replicate findings and discover new IVs in the participant populations, even enabling the identification of association signals due to rare variants.

The studies included in multi-ethnic meta-analyses must satisfy certain requirements in terms of sample size, extent of genotyping, and phenotyping. Therefore, this is a route that not every Latin American study may be able to follow. For the most part, multi-ethnic meta-analyses at the moment consist of individuals of European descent, with individuals of other ancestries accounting for a much smaller proportion. 

## 3. Widening the Net: Following Manolio’s Suggestion [13]

An alternative to a multi-ethnic study as described above is to extend the analysis of research projects involving a primarily European population to include non-European samples that were also collected and genotyped [13]. Non-European participants’ data are frequently ignored, in an attempt to guard from confounding caused by population stratification or due to limited statistical power as a result of lower subject numbers. As statistical methods improve, studies embracing all ethnicities become easier to perform, and limitations are overcome to produce a better picture of disease risk across the world. The inclusion of subjects of Latin American origin via this route might occur more often in studies carried out in the United States, as individuals of this origin make up approximately 18% of the population (https://www.pewresearch.org/hispanic/fact-sheet/latinos-in-the-u-s-fact-sheet/). Nevertheless, it should be mentioned that there are several ongoing studies in the US that have prioritized the collection of data on Hispanic populations and have recruited considerable numbers of Latinos, including (but not limited to) the Women’s Health Initiative (WHI, https://www.whi.org/SitePages/WHI%20Home.aspx), the Study of Latinos (SOL, https://sites.cscc.unc.edu/hchs/), the Multi-ethnic Study of Atherosclerosis (MESA, https://www.mesa-nhlbi.org/), and the Multiethnic Cohort (MEC, https://www.uhcancercenter.org/mec). The Population Architecture using Genomics and Epidemiology (PAGE) study (https://www.pagestudy.org/), which comprises two of these cohorts plus the Icahn School of Medicine at Mount Sinai BioMe biobank, is dedicated to increasing representation of ancestrally diverse populations living in the US in genetic epidemiology research [14].

However, given that the largest and more sustained migratory waves of Latin Americans to the US stemmed predominantly from Mexico, Puerto Rico, and Cuba, these populations are far likelier to be represented in research studies, as opposed to other groups for which migration north is difficult or not appealing, or which come from countries with smaller population sizes. Later considerable migration from Central American countries such as Honduras, El Salvador, and Guatemala, or South American countries, like Venezuela, may provide additional diversity to multi-ethnic studies if their citizens (and their descendants) become part of these research efforts. All the same, it should be kept in mind that Latinos living abroad are less likely to experience the same environmental exposures as at home, and that individuals who migrate may not be representative of their source population.

## 4. Participating in Large Regional Consortia

A further route is the creation of regional or national consortia, which allow for a more exhaustive assessment of the populations of interest. Because sample sizes are larger (although not as large as those in some biobanks, for instance), risk polymorphisms identified in European populations can be validated; and new genetic risk factors can also be discovered this way. Several of the existing consortia are disease-based, for example, those investigating breast cancer (see Zavala and colleagues in this special issue for a few examples [15], or the upcoming LAGENO-BC: Latin American Genetics/Genomics of Breast Cancer Consortium), lung cancer (CLICAP: Consorcio Latino Americano para la Investigación del Cáncer de Pulmón, http://clicap.info/index.php/) and colorectal cancer (CHIBCHA: Genetic study of Common Hereditary Bowel Cancers in Hispania and the Americas, https://cordis.europa.eu/project/id/223678), while some include population-based cohorts with plenty of epidemiological data (e.g., EPIGEN-Brasil: https://epigen.grude.ufmg.br/index.php), and others are oriented preferentially to population genetics (e.g., CANDELA: Consortium for the Analysis of the Diversity and Evolution of Latin America, https://www.ucl.ac.uk/candela; Chile Genómico: http://www.chilegenomico.cl/).

Consortia pose problems of their own, with issues like data harmonization, difficulties sharing data across consortia and with the wider scientific community, and data archiving representing hurdles to their optimal functioning [16].

From a MR perspective, availability of phenotypes of modifiable exposures is vital, as the aim is not quite to identify genetic risk factors of disease but to determine the causality of environmental and lifestyle risk factors using a genetic tool. Therefore, consortia that are short of this type of data on control individuals will not be as suitable for this endeavor.

## 5. Generating New Information Locally: Genome-Wide Association and Sequencing Studies in Latin American Populations

The best-case scenario would be performing GWAS, linkage analyses, admixture mapping, or sequencing studies in the populations of interest, as these are hypothesis-generating methods that require large numbers of subjects in order to reach enough statistical power to detect an effect and obtain precise estimates. This approach allows not only the validation (or not) of already identified IVs, but most importantly, the discovery of new IVs of relevance in Latin America. In fact, an admixture mapping study carried out on participants of the EPIGEN-Brasil consortium recently reported a low frequency variant of chromosome 13 strongly associated with body mass index (BMI) in females [17]. This variant, very rare in Europeans, exhibited a notably larger effect size in Brazil than that of SNPs in fat mass and obesity-associated gene (*FTO*), one of the genes with the most compelling association amongst Europeans. Another example, a GWAS using the CANDELA consortium, which includes over 6000 individuals recruited in Brazil, Chile, Colombia, Mexico, and Peru, showed a robust association between a missense variant in *MFSD12* and skin pigmentation. The variant is frequent in Native Americans and East Asians, and the gene has been associated with pigmentation in Africans, but not in Europeans [18]. Other GWAS have been completed in Brazil [19], Chile [20], and Mexico [21] of traits as different as asthma and atopy, gallstone disease, and BMI and body fat.

On the other hand, this strategy requires considerable recruitment and funding efforts, which are out of reach for many Latin American countries. It is more achievable in Hispanic/Latinos living in the US, and there are numerous published examples (e.g., [22,23]).

A public–private initiative to sequence the genome of 15,000 Brazilians from the Estudo Longitudinal de Saúde do Adulto (ELSA-Brasil, http://www.elsa.org.br/oelsabrasil.html [24]) study is currently underway, called *DNA do Brasil.* It is particularly interesting because there are already a lot of epidemiological data on ELSA participants, and the addition of genetic information will generate a coveted resource for genetic epidemiologists. 

## 6. Validating European-Identified Instrumental Variables in Regional Populations

When running large-scale studies in a local population is not possible, there is the option of trying to validate genetic variants associated with the risk factor of interest in Europeans. Most contemporary Latin American populations originated from the admixture between Native Americans, Europeans, and Africans; thus, they may have inherited genetic risk factors from these parental populations. Examples of this pathway can be found in Mexico, where SNPs associated with BMI in Europeans were tested in Mexican children and adults [25,26]. European admixture proportions vary along the continent, as do the European parental populations, i.e., Portuguese, Spanish, and French. Considering the genetic variants that are risk factor IVs in these distinctive parental groups might be a good start. In addition, genetic variants associated with exposure in African or East Asian populations could also be prioritized for validation in Latin American populations with significant African or Native American ancestry, respectively.

## 7. Making Sense of Small Local Studies

There has been a lot of candidate gene research carried out in Latin America (attested by several of the articles published in this Special Issue [27,28,29,30,31]), possibly because resources are limited, and having a clear hypothesis of the relationship between genetic variation and a specific outcome allows the use of sample sizes within the realm of possibilities for regional research groups. This important research is not fully taken advantage of, as it is difficult to merge its results together in meta-analyses. Some of the reasons for this are that findings are presented in many different ways, and missing information varies between studies. Meta-analyses are useful tools for the synthesis of results across individual studies and for identifying gaps in evidence, but they have a few limitations that may make their findings unreliable, such as small study effects, publication bias, poor quality of primary studies, and high heterogeneity between studies. However, established guidelines and methodological developments (when available) can be implemented to produce meta-analyses as informative as possible [32]. 

As an illustration, and with the caveat of not being a formal systematic review and meta-analysis, we have compiled all data we could find on the association of the fat mass and obesity-associated gene (*FTO*) polymorphism rs9939609 with body mass index (BMI) and obesity in the Brazilian population, by performing a PubMed and LILACS search (for *FTO* and Brazil; and rs9939609 and Brazil), and searches for *FTO* and rs9939609 in the Biblioteca Digital Brasileira de Teses e Dissertações (http://bdtd.ibict.br/vufind/). Additionally, we considered papers that investigated the association of rs9939609 with other traits in order to extract the allele frequency of this SNP in the particular Brazilian population under study. *FTO* (and SNP rs9939609) was one of the first genes to be detected as strongly associated with BMI in GWAS performed in populations of European descent [33], and has been replicated numerous times, including in non-European populations (see GWAS catalog: https://www.ebi.ac.uk/gwas/). The A allele is associated with an increase in BMI or the risk of obesity. The average frequency of the A allele in Europe is 0.414, in populations of the Americas it is 0.262, and in African populations it is 0.494, according to the 1000 Genomes database (https://www.internationalgenome.org/home).

**Figure 1:**
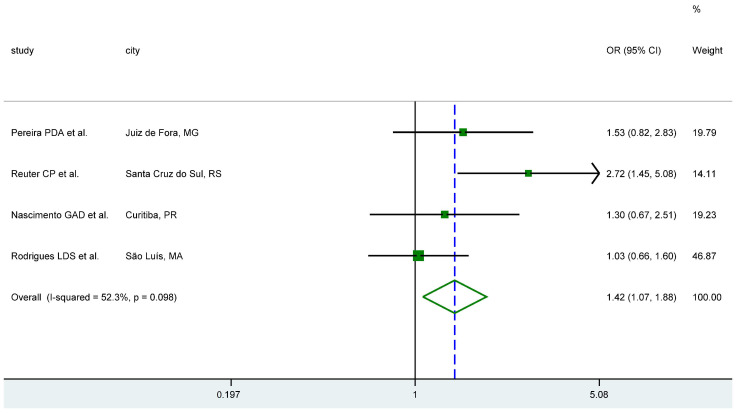
Effect of SNP rs9939609 (*FTO*) on being overweight or obese amongst Brazilian children and adolescents with the AA genotype vs those with the TT genotype [34,35,36,37].

We uncovered 44 studies across Brazil, which used 35 different population samples. Most of the studies were done on women and children/adolescents (*n* = 15 each), while 12 were done on special groups, such as individuals who underwent bariatric surgery, or diabetes patients (Supplementary Table S1). Evidence of association of rs9939609 with BMI or obesity was weak throughout. We ran a fixed-effects meta-analysis of four studies, comparing genotypic frequencies between normal weight and overweight/obese children and adolescents (as defined by the World Health Organization, https://www.who.int/en/news-room/fact-sheets/detail/obesity-and-overweight), which included 1873 individuals (964 normal weight and 909 overweight/obese). We found that individuals who were homozygotes AA had 1.42 times (95% CI 1.07, 1.88) greater odds of being overweight or obese than individuals who were homozygotes for the T allele (Figure 1). This result is fairly similar to what was reported by Frayling et al. in their analysis of European populations [33]. Meta-analysis results for the TA vs. TT and AA vs. TA/TT comparisons are shown in Supplementary Figures S1 and S2. We also ran a fixed-effects meta-analysis of four studies of adults that reported mean BMI in kg/m^2^ by genotype (N = 1420). Results in this case showed no evidence of association with rs9939609 (Supplementary Figure S3).

**Figure 2:**
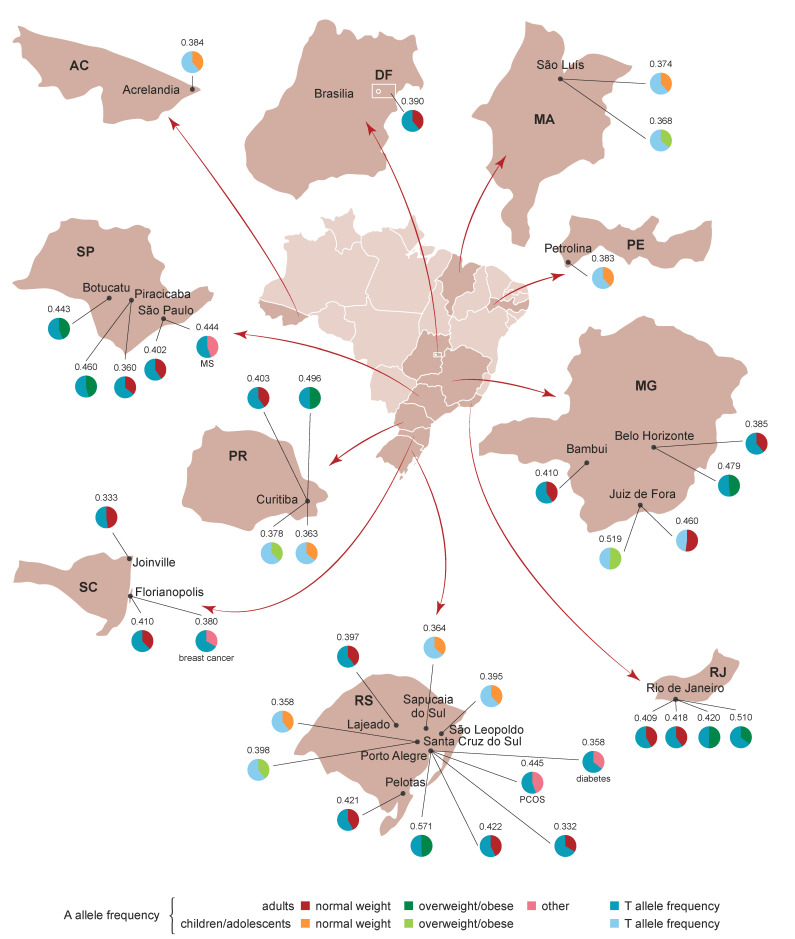
Map of Brazil depicting the allele frequency distribution of *FTO* rs9939609. States and cities where the studies were carried out are shown. For studies using the same population, average allele frequencies are given. State names: AC = Acre, DF = Distrito Federal, MA = Maranhão, MG = Minas Gerais, PR = Paraná, RJ = Rio de Janeiro, RS = Rio Grande do Sul, SC = Santa Catarina, SP = São Paulo. MS = metabolic syndrome. PCOS = polycystic ovary syndrome.

Allele frequencies in populations around the country did not diverge much and were close to those reported for European and other American populations (except Peruvians, with an A allele frequency of 0.082). The weighted average frequency of the A allele for different subgroups is shown in Table 1. The distribution of rs9939609 allele frequencies in Brazilian populations is depicted in Figure 2.

Some conclusions can be readily extracted from this succinct analysis. First, while several studies examined the relationship between *FTO* and BMI or obesity, there were quite a few that investigated other outcomes, interactions, and patient populations, in certain cases assuming an underlying *FTO*–BMI association. Second, regression analysis of this relationship was infrequent; therefore, so was adjustment for covariates that may confound it. Third, there was limited mention of confounding by population stratification, despite the well-known admixed background of Brazilians; nonetheless, some studies attempted to take this into account, adjusting for skin color, race, or ethnicity. Only a couple of studies ran models adjusted for principal components [38] or genetic ancestry [17]. Fourth, there is a very uneven, yet not unexpected, distribution of studies throughout the country, with concentration on the south and southeast regions, and on women and children. Overall, from the IV point of view, the picture that emerges is inconclusive, although there is indication of an association of rs9939609 with obesity in children.

Unfortunately, because of the disparities between studies, including more of them in meta-analyses was not possible. As a consequence, we would like to propose that authors report findings more thoroughly, by submitting as much detail as possible on the information listed in Table 2, by way of Supplementary Materials if necessary. It would also be desirable to contemplate expanding the investigation of the genetic determinants of BMI (and other traits) outside of the southernmost states of Brazil, and incorporating samples of individuals with a wide spectrum of demographic characteristics. Doing this will help generate evidence on the likelihood of a genetic polymorphism acting as an IV for a risk factor in a specified population, even though we may not be able to meaningfully use the effect estimates from small local studies in a MR conventional analysis. Even so, once the association of the IV with the risk factor is more firmly established, future studies of the association of the genetic variant with an outcome would provide a test for an effect of the proxied exposure on the outcome, if the exclusion-restriction assumption holds [39,40].

## 8. Conclusions

We have offered a brief assessment of the potential of using genetic data from Latin American populations in future studies employing MR or other similar genetically informed methods of causal inference. For MR to work, strong instruments for the exposures of interest are required; hence, we believe this should be our initial focus. Latin America is a large, diverse region, which includes 20 countries and 14 dependent territories, and is home to almost 645 million people. In addition, there are ~60 million Hispanic/Latinos presently living in the US. Although Latin American populations share a complex genetic background emerging from past conquest and colonization that brought together indigenous peoples, European settlers, and enslaved Africans, each has also experienced a distinct history of admixture and adaptation.

No study will be able to capture this diversity in its entirety, so unravelling the genetic basis of Latin American disease risk factors will be like putting a puzzle together, and each piece helps. Studies that can run GWAS or do whole-genome or exome sequencing will be highly valuable, as well as those that can become part of a multi-ethnic meta-analysis or a regional consortium, but the contribution of smaller studies within countries, in particular those where the former are not an option, is important too. Besides supplying evidence for the association of the genetic instrument with the risk factor, they are likely to contribute evidence of pleiotropic associations and associations with confounders, which may be sources of bias in forthcoming MR studies. In order to extract the maximum benefit, we must ensure the data are adequately reported and can be safely and rapidly shared, harmonized, and analyzed by collaborating researchers across the continent. While difficult to put together for practical and economic reasons, even larger initiatives, like the UK Biobank (https://www.ukbiobank.ac.uk/) or the China Kadoorie Biobank (https://www.ckbiobank.org/site/), could potentially be replicated in Latin America’s largest countries, of which Mexico is leading the way (https://mxbiobankproject.org/). Combining these efforts and subsequently adding the smaller countries would generate a major resource for the region’s public health system, which would be worth investing in.

## Figures and Tables

**Table 1:** Weighted frequency of rs9939609 A allele in subgroups of the Brazilian population.

Subpopulation	A Allele Frequency	N
normal weight children	0.374	3762
overweight/obese children	0.402	1148
normal weight women	0.375	1926
overweight/obese women	0.455	847

**Table 2:** Useful information to be reported from local studies.

Type of Information	Number of Studies ^a^
type of study	37
Hardy–Weinberg equilibrium	42
effect allele	44
DNA strand for palindromic SNPs	0
allele frequencies/counts	41
genotypic frequencies/counts	27
participants’ ethnicity	27
total N	43
OR and 95% CI/SE	13
beta coefficients and 95% CI/SE	7
covariates adjusted for	15
correction for population stratification	12

^a^ Total number of studies from our search = 44.

## Supplementary Materials

The following are available online at https://www.mdpi.com/2073-4425/11/5/507/s1, Figure S1. Effect of SNP rs9939609 (*FTO*) on being overweight or obese amongst Brazilian children and adolescents with the TA genotype vs. those with the TT genotype. Figure S2. Effect of SNP rs9939609 (*FTO*) on being overweight or obese amongst Brazilian children and adolescents with the AA genotype vs. those with TA or TT genotypes. Figure S3. Effect of SNP rs9939609 (*FTO*) on BMI in kg/m2 amongst Brazilian adults with the AA genotype vs. those with the TT genotype, Table S1: Studies that examined *FTO* rs9939609 and BMI or obesity in Brazil.
